# Poor hygiene of housing conditions influences energy metabolism in a muscle type-dependent manner in growing pigs differing in feed efficiency

**DOI:** 10.1038/s41598-022-12050-6

**Published:** 2022-05-14

**Authors:** Annie Vincent, Frédéric Dessauge, Florence Gondret, Bénédicte Lebret, Nathalie Le Floc’h, Isabelle Louveau, Louis Lefaucheur

**Affiliations:** PEGASE, INRAE, Institut Agro, 35590 Saint-Gilles, France

**Keywords:** Metabolism, Animal physiology

## Abstract

The ability of pigs to cope with inflammatory challenges may by modified by selection for residual feed intake (RFI), a measure of feed efficiency. In the current study, we evaluated skeletal muscle metabolic responses to degraded hygiene conditions in pigs divergently selected for RFI. At 82 d of age, low RFI and high RFI pigs were housed in either poor or good hygiene conditions. After a 6-week challenge, the poor hygiene conditions induced a decrease in growth performance (*P* < 0.001) and in plasma IGF-I concentrations (*P* < 0.003) in both lines. In the slow-twitch oxidative *semispinalis* muscle, poor hygiene conditions induced a shift towards a more oxidative metabolism and an activation of the AMPK pathway in pigs of both RFI lines. In the fast-twitch glycolytic *longississimus* muscle, poor hygiene conditions were associated to a less glycolytic metabolism in the HRFI line only. Poor hygiene conditions also increased the protein level of lipidation of microtubule-associated protein 1 light-chain 3β (LC3-II) in both RFI lines, suggesting an activation of the autophagy pathway. Altogether, the data revealed muscle-type specific metabolic adaptations to poor hygiene conditions, which may be related to different strategies to fuel the activated immune system.

## Introduction

Pigs may face non-optimal sanitary conditions during rearing periods. Low sanitary conditions impair pig health and induce a systemic inflammation that in turn alters metabolism and reduces growth performance^1,2^. The interplay between inflammation and metabolism is further illustrated by changes in the blood metabolome including amino acids, energy-yielding metabolites, and DNA/RNA metabolism in response to pig sanitary status^3,4^. Furthermore, inflammation resulting from Lipopolysaccharide (LPS) challenge has been reported to modify glucose metabolism and mitochondrial function in pig muscle^5^. Coping with sanitary stress requires substantial amounts of energy to activate the immune system. The ability of pigs to reallocate energy and nutrients toward this system may be affected by selection for feed efficiency^6^. Independent divergent selection experiments for residual feed intake (**RFI**) in growing pigs submitted to an immune challenge based on poor hygiene of housing conditions^7^ or to viral or bacterial infections^8,9^ revealed that growth rate and feed efficiency were less altered in the low RFI line (**LRFI**, i.e. high feed efficiency) when compared with the high RFI line (**HRFI**, i.e. low feed efficiency). The two lines coped with the inflammatory challenge using different metabolic strategies^4^. Substantial differences between RFI lines in gene expressions related to defense, immune pathway, oxidative stress and protein metabolism have been found in different tissues, including muscle^10,11^. Importantly, the expressions of genes related to glycogen utilization were specifically up-regulated in the *longissimus* muscle, a white fast-twitch glycolytic muscle, in pigs from the LRFI line (more efficient), whereas genes related to fatty acid oxidation were down-regulated in LRFI pigs compared with HRFI pigs^10^. In pigs facing poor hygiene of housing conditions, there was a short-term preservation of muscle weight in both lines, but muscle growth rate was reduced more specifically in HRFI pigs during the post-challenge period^12^, suggesting that the early muscle adaptation to hygiene conditions may depend on the RFI line and could influence the subsequent animal growth.

Among factors regulating energy pathways in muscle, the adenosine monophosphate 5’-AMP-activated protein kinase (**AMPK**) has been identified as a key sensor of cellular energy deficit to regulate energy balance in skeletal muscle^13,14^. In response to energy depletion, phosphorylated AMPK at Thr172 inhibits anabolic pathways and stimulates catabolic ones to restore cellular energy level. In addition to this well-characterized role of AMPK in the regulation of nutrient metabolism, it is increasingly clear that AMPK activation has also multiple actions on inflammatory signaling^15^. Noteworthy, an inhibition of the mammalian target of rapamycin (**mTOR**) pathway acting as an intracellular nutrient sensor to control protein synthesis, cell growth and metabolism, has been also reported after a severe viral infection^16^, which could explain the reduction in muscle protein synthesis observed in response to severe inflammation. Importantly, mTOR is one of the downstream targets of AMPK functions, and the AMPK and mTOR signaling pathways interplay in regulating energy balance^17^. Both pathways also regulate autophagy^18^, an intracellular degradation process^19^. Furthermore, skeletal muscles with different contractile and metabolic properties may exhibit different responses to nutritional stimuli, as previously reported in growing piglets fed a deficient diet in total sulfur amino acid^20^. The metabolic strategies used by muscles of different types and from different RFI lines to cope with an inflammatory challenge remain to be clarified.

To our knowledge, there are few available data regarding the biological changes in skeletal muscles of growing pigs facing moderate inflammation as encountered in pig production. Therefore, the current study was undertaken to determine whether the inflammation induced by poor hygiene of housing conditions during the growing phase of pigs differing in RFI background may impact metabolic pathways in two skeletal muscles differing in contractile and metabolic properties. Both AMPK and mTOR pathways and hormones playing a significant role in metabolic regulations were investigated.

## Materials and methods

The experiment was performed in the INRAE UE3P experimental facility at Saint-Gilles (10.15454/1.5573932732039927E12) in accordance with the ethical standards of the European Community (Directive 2010/63/EU), and was approved by the regional ethics committee in animal experimentation (Comité Rennais d’Ethique en matière d’Expérimentation Animale, CREEA, number 07). This study is reported in accordance with ARRIVE guidelines (https://arriveguidelines.org).

### Animals, housing and experimental design

The trial was performed on a subset of 32 pigs (16 males, 16 females) among the first batch period 1 of a larger study previously described^7^. Pigs originated from the 8th generation of a divergent selection for RFI^21^. They were born in the INRAE experimental facility and were weaned at 4 weeks of age. At 12 weeks of age (26.9 ± 2.7 kg BW), pairs of littermates (same sex: males or females) exhibiting similar BW were housed in individual pens (85 × 265 cm) on concrete floor throughout the 6-week experimental period. Within each pair, animals were assigned to either good or poor hygiene conditions. The experimental housing conditions applied to pigs have been previously described^7^. Briefly, poor hygiene conditions were obtained by not cleaning the room after a previous occupation by non-experimental pigs. Conversely, good hygiene conditions were obtained after room cleaning and disinfection, in addition to the application of optimal aeration rate and temperature and strict biosecurity precautions.

Animals had free access to a standard cereal and soybean meal-based growing diet (13.17 MJ of digestible energy/kg; 153 g of crude protein/kg; tryptophan/lysine 0.2; lysine 8.3 g/kg; dry matter 87.38%; starch 44.17%; neutral detergent fiber 15.7%; acid detergent fiber 5.6%; acid detergent lignin 1.6%; fat 3.14%; wheat 32.3%; barley 30%; maize 15%; soya bean 7%; bran 5%) and to water. Feed consumption per pig was estimated weekly as the difference between allocated feed minus feed refusals. This allowed to calculate average daily feed intake (ADFI). Pigs were weighed after an overnight fast at the beginning and at the end of the sanitary challenge. The trial duration was 6 weeks. At the end of the trial (i.e., at 18 weeks of age), the pigs were euthanized by electrical stunning and exsanguination. At bleeding, blood samples (10 mL) were collected on EDTA and centrifuged for 15 min at 2500 g at 4 °C. Plasma was stored at − 20 °C until further analyses.

### Muscle chemical composition and metabolic enzyme activities

A few minutes after exsanguination (time T0), samples (about 10 g) of *longissimus* muscle (LM) at the last rib level and of *semispinalis* muscle (SM) at the neck level were taken, immediately frozen in liquid nitrogen and stored at − 80 °C until further characterization by western blot analyses. Additional samples of LM and SM were collected 30 min after slaughter (T30), frozen in liquid nitrogen and stored at  − 80 °C until further analyses. The glycolytic potential (GP = 2 ([glycogen] + [glucose] + [glucose-6-phosphate]) + [lactate]) was measured as a marker of muscle glycogen concentration at slaughter. Activities of lactate dehydrogenase (LDH), β-hydroxy-acyl-CoA dehydrogenase (HAD), and citrate synthase (CS) were also determined as markers of the glycolytic, fatty acid β-oxidation, and terminal oxidative capacities, respectively. The GP and metabolic enzyme activities were assessed according to procedures previously outlined^22^. At 24 h postmortem, additional LM and SM samples were trimmed of external fat, minced and freeze-dried to determine dry matter and protein contents^23^ and intramuscular fat content by the application of supercritical CO_2_ and solvent extraction with an automatic system (Leco TFE 2000 Instrument, Leco, St. Joseph, MI)^12^.

### Muscle metabolic and autophagy pathway analyses

Muscle homogenates (30 µg of proteins) were separated by SDS-PAGE (sodium dodecyl sulfate–polyacrylamide gel electrophoresis), electro-transferred to polyvinyllidene difluoride membranes and incubated overnight at 4 °C with corresponding primary antibodies as described previously^24^. Antibodies used were total AMPKα (Cell Signaling, #2793, 1:2000), phosphorylated pAMPKα Thr172 (Cell Signaling, #2535, 1:1000), total mTOR (Cell Signaling, #2983, 1:1000), phosphorylated pmTOR Ser2448 (Cell Signaling, #5536, 1:1000), microtubule-associated protein 1 light-chain 3β (MAP LC3 β ; Santa Cruz Biotechnology, sc-271625, 1:1000), and the mitochondrial subunit COX IV (Cell Signaling, #4850, 1:1000). After five washes in TBST, membranes were incubated with the appropriate horseradish peroxidase-conjugated secondary antibodies for 1 h at room temperature. After five washes in TBST, blots were developed by using an enhanced chemiluminescence kit (GE Healthcare Sciences), scanned with an ImageQuant LAS 4000 system (GE Healthcare) and analyzed with the ImageQuant TL programm. Bands were quantified as arbitrary units (AU) after standardization to a reference sample added on each gel, which made comparisons between membranes relevant for a given antibody. The ratio of phosphorylated to total AMPK or mTOR was determined for each sample.

### Plasma hormone concentrations

All samples were analyzed in duplicate in a single batch. Plasma total triiodothyronine (TT3) and total thyroxin (TT4) concentrations were determined using commercial immunoassay kits (ST AIA-PACK T3 or T4 respectively, TOSOH) and the AIA-1800 device (Automated Immunoassay Analyzer; TOSOH). The intra-assay CV were less than 7% for the two hormones. Plasma leptin concentrations were quantified using the multispecies radioimmunoassay kit (XL-85 K; Immunodiagnostic System) previously validated for use in porcine plasma^25^. The intra-assay CV was 3.1% for a leptin concentration of 7 ng/mL. Plasma concentrations of total ghrelin were quantified using a RIA kit (Phoenix Pharmaceuticals, Inc.) that has been used previously in pigs^26^. The intra-assay CV was 8% at 220 pg/mL. Plasma IGF-I concentration was determined after an acid–ethanol extraction using the IRMA IGF-I kit (Beckman-Coulter-Immunotech). The intra-assay CV was 12% at 553 ng/mL.

### Statistical analyses

Statistical analyses were carried out using the SAS software (SAS Institute, Cary NC, New-York). First, statistical analyses were carried out within each muscle type using three-way ANOVA including hygiene conditions (Good or Poor), line (LRFI or HRFI), sex (male or female) and their interactions as main effects. Because there was no sex effect for the studied traits, the analyses were finally run using two-way ANOVA including hygiene conditions, line and the interaction between hygiene conditions and line. Pig was considered as the experimental unit. Initial BW was included in the model as a covariate to analyze growth performance. Least square means were compared using the LS means statement and the pdiff option of the GLM procedure. All data are reported in tables as LS means per experimental group, and a pooled SEM per trait. Differences were considered significant when *P* < 0.05, and 0.05 ≤ *P* < 0.10 was discussed as a trend.

## Results

### Brief summary of growth performance under challenged Conditions

**Figure 1:**
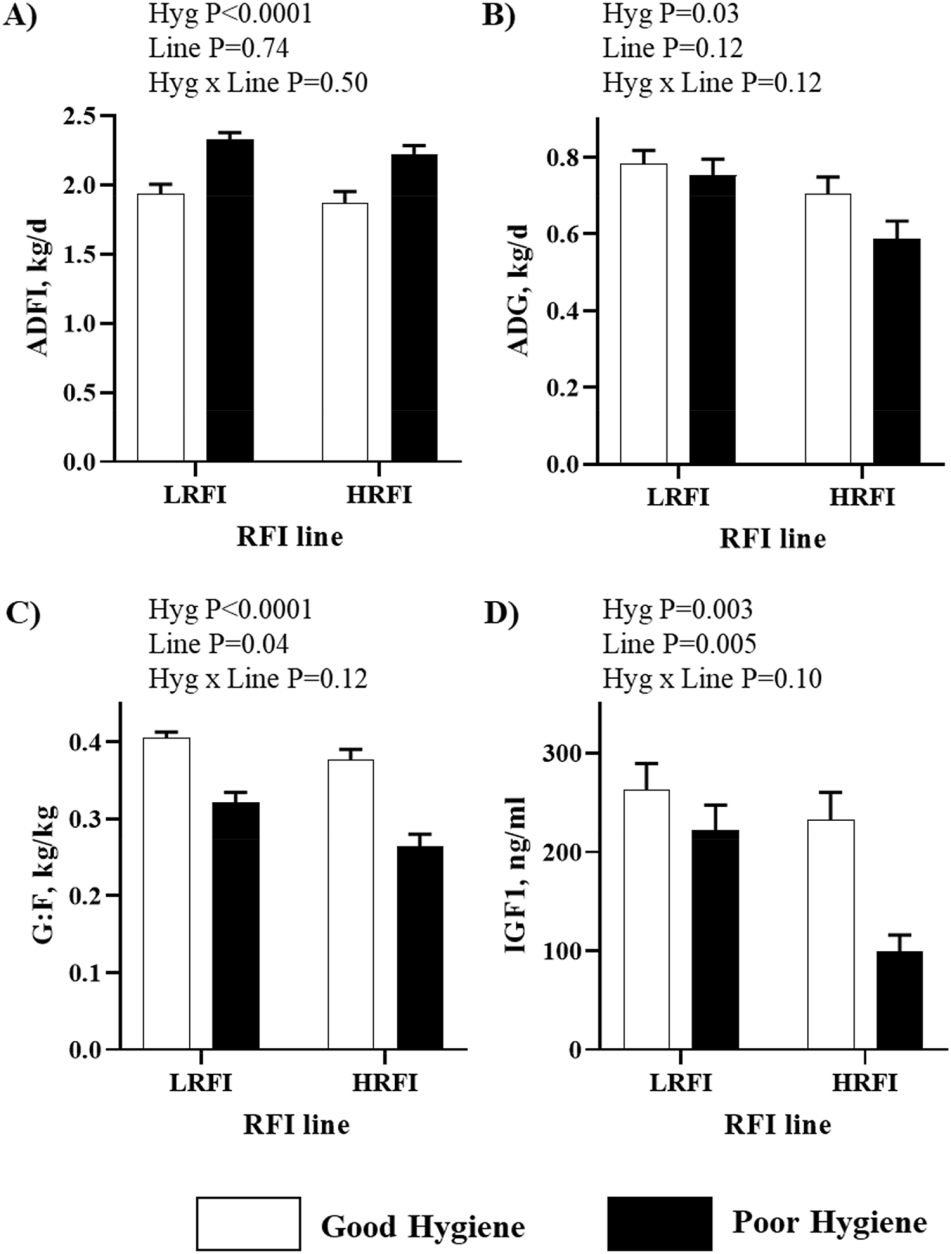
Effect of poor versus good hygiene condition (Hyg) on growth performance and plasma IGF-I concentration of pigs divergently selected for low or high residual feed intake (LRFI and HRFI lines). (**A**) Average daily feed intake (ADFI), (**B**) Average daily gain (ADG), (**C**) Gain to feed ratio (G:F), and (**D**) Insulin-like growth factor-I (IGF1). Means ± standard error of the mean are reported on figures (n = 6 to 10/experimental group).

The full experimental design and results on animal growth performance have been previously described^7^. Consequences on growth performance of LRFI and HRFI pigs after exposition to poor or good hygiene conditions during 6 weeks in the growing period are thus only briefly summarized for the subset of pigs considered in the present study (n = 32). All pigs were killed at 124.7 ± 2.2 d. There was no interaction between housing conditions and RFI lines for any growth traits investigated. As shown in Fig. 1A, ADFI was increased in poor hygiene conditions compared with good hygiene conditions for both lines (+ 19%, *P* < 0.001). Compared with good hygiene conditions, poor hygiene conditions led to reduced pig average daily gain (*P* < 0.05; Fig. 1B) and gain to feed ratio (G:F; *P* < 0.001; Fig. 1C) during the 6-week experimental period in both RFI lines. As expected, HRFI pigs exhibited a lower G:F ratio than LRFI pigs during the experimental period (*P* < 0.05).

### Hormonal status

Fasting plasma concentrations of IGF-I at the end of the sanitary challenge were reduced in pigs housed in poor hygiene conditions compared with pigs housed in good hygiene conditions (*P* < 0.01, Fig. 1D). It was also lower in HRFI than in LRFI pigs (*P* < 0.01). Fasting plasma concentrations of total T3, total T4, leptin and ghrelin did not differ significantly between the four experimental groups (Table 1).

**Table 1:** Plasma hormone concentrations of pigs divergently selected for low (LRFI) or high (HRFI) residual feed intake and raised in good or poor hygiene conditions (Hyg).

Item	LRFI		HRFI		SEM	*P* values		
	Good	Poor	Good	Poor		Hyg	Line	Line x Hyg
N	9	10	7	6				
TT3, ng/dl	80.20	86.30	76.24	74.17	14.56	0.71	0.14	0.44
TT4, µg/dl	4.28	4.18	4.03	4.06	0.79	0.90	0.51	0.82
Leptin, ng/ml	4.02	4.28	3.81	4.01	0.67	0.35	0.33	0.90
Ghrelin, pg/ml	239.0	268.3	268.7	326.3	104.3	0.26	0.25	0.71

Plasma total triiodothyronine (TT3) and total thyroxin (TT4) concentrations were determined using commercial immunoassays. Plasma concentrations of leptin and ghrelin were quantified using radioimmunoassay. Values are LS means.

### Muscle chemical composition and metabolism

The chemical composition of LM and SM did not differ significantly between the two housing conditions but was influenced by the RFI line. In LM, dry matter and IMF content were greater (*P* < 0.05) in HRFI than in LRFI pigs (Table 2), whereas GP was lower in HRFI pigs than in LRFI pigs (*P* = 0.01). In SM, the protein content tended (*P* < 0.10) to be lower in HRFI than in LRFI pigs. There was a Hyg x Line interaction (*P* = 0.01) for GP, which was lower in HRFI pigs housed in good hygiene conditions compared with HRFI pigs housed in poor conditions, whereas the two groups of LRFI pigs had a similar GP.

**Table 2:** Chemical composition of *longissimus* and *semispinalis* muscles of pigs divergently selected for low (LRFI) or high (HRFI) residual feed intake and raised in good or poor hygiene conditions (Hyg).

Item	LRFI		HRFI		SEM	*P* values		
	Good	Poor	Good	Poor		Hyg	Line	Line x Hyg
N	9	10	7	6				
*Longissimus*								
Dry matter, %	24.11	24.20	24.55	24.60	0.33	0.56	0.004	0.88
Protein, %	21.48	21.66	21.90	21.80	0.49	0.71	0.17	0.44
IMF, %	0.80	0.70	0.96	1.12	0.31	0.96	0.02	0.26
GP, µmol/g	206.2	209.1	175.5	192.0	22.1	0.30	0.01	0.58
*Semispinalis*								
Dry matter, %	25.19	25.18	25.11	25.14	1.29	0.99	0.89	0.96
Protein, %	18.17	17.74	17.64	17.69	0.44	0.25	0.08	0.14
IMF, %	4.94	5.35	5.63	5.35	1.30	0.89	0.47	0.47
GP, µmol/g	127.1^b^	114.2^b^	87.5^a^	113.8^b^	20.4	0.37	0.02	0.01

Intramuscular fat (IMF) was expressed as percentage of fresh muscle and glycolytic potential (GP) as micromoles of equivalent lactate per gram of fresh muscle. Values are LS means. ^a,b^ Within a row, means without a common superscript differ (P < 0.05).

Hygiene conditions modified the energy-related enzyme activities in a muscle-dependent manner. In LM, interactions between hygiene condition and line were significant (*P* ≤ 0.05) for LDH and LDH/CS ratio, with a decreased activity and ratio in poor HRFI pigs compared with good HRFI pigs and no variation with hygiene conditions for the LRFI line, which had values similar to poor HRFI pigs (Table 3). Activities of CS and HAD and levels of COX IV did not differ significantly according to hygiene conditions or RFI lines in LM. In SM, all measured enzymes were affected by hygiene of housing conditions but were not significantly influenced by the RFI line. Activities of CS and HAD and the abundance of COX IV were higher (*P* < 0.05) in pigs housed in poor conditions than in good conditions. Poor hygiene conditions also tended to decrease LDH activity in SM (*P* < 0.10). Consequently, the LDH/CS ratio in the SM was drastically reduced (− 30%, *P* < 0.001) in pigs from poor hygiene conditions compared with pigs from good hygiene conditions.

**Table 3:** Activities of lactate dehydrogenase (LDH), citrate synthase (CS), and β-hydroxy-acyl-CoA dehydrogenase (HAD) and levels of COX IV in *longissimus* and *semispinalis* muscles of pigs divergently selected for low (LRFI) or high (HRFI) residual feed intake and raised in good or poor hygiene conditions (Hyg).

Item	LRFI		HRFI		SEM	*P* values		
	Good	Poor	Good	Poor		Hyg	Line	Line x Hyg
N	9	10	7	6				
*Longissimus*								
LDH	1948^a^	2094^a^	2652^b^	1952^a^	452	0.10	0.10	0.01
CS	6.35	6.89	6.95	6.97	0.73	0.30	0.21	0.32
HAD	4.01	4.46	4.55	4.34	0.47	0.48	0.23	0.07
LDH/CS	310.9^a^	306.8^a^	377.8^b^	281.4^a^	61.6	0.03	0.36	0.05
HAD/CS	0.64	0.65	0.66	0.63	0.09	0.86	0.90	0.47
COX IV, AU	4.26	4.82	4.66	5.15	0.98	0.14	0.32	0.92
*Semispinalis*								
LDH	510	473	478	414	74	0.07	0.14	0.62
CS	13.79	16.45	12.19	15.93	1.53	< 0.001	0.07	0.33
HAD	11.40	13.32	9.82	14.13	2.22	< 0.001	0.64	0.15
LDH/CS	37.4	28.8	39.9	26.2	6.8	< 0.001	0.97	0.32
HAD/CS	0.84	0.81	0.81	0.87	0.12	0.74	0.67	0.30
COX IV, AU	7.56	9.28	7.74	8.18	1.12	0.012	0.27	0.12

Enzyme activities were expressed as micromoles of substrate degraded per minute per gram of fresh muscle. COX IV was determined by western blot and expressed as arbitrary units (AU). Values are LS means. ^a,b^ Within a row, means without a common superscript differ (P < 0.05).

### AMPK, mTOR and autophagy pathways

In LM, total AMPK and pAMPK levels as well as the pAMPK/AMPK ratio were not influenced by hygiene conditions. The level of total AMPK was greater (*P* = 0.01) but pAMPK tended to be higher (*P* = 0.07) in HRFI than LRFI pigs, so that the pAMPK/AMPK ratio was similar in both lines (Table 4). The abundance of total mTOR and pmTOR were unaffected by the hygiene conditions nor by RFI line. Two specific LC3 bands were observed in blots at 16 kDa for LC3-I and at 14 kDa for LC3-II, respectively. The abundance of LC3-II was increased by the poor hygiene conditions (*P* < 0.05) in both RFI lines. In SM, poor hygiene conditions led to an increased abundance in total AMPK and pAMPK (*P* < 0.05) and tended to increase pAMPK/AMPK. Total mTOR was also increased by poor hygiene conditions (*P* = 0.01). Irrespective of hygiene conditions, total AMPK and pAMPK were lower (*P* < 0.05) in HRFI than LRFI pigs, leading to similar pAMPK/AMPK in both lines. LC3-I and LC3-II were not affected by hygiene conditions or RFI line in SM.

**Table 4:** Protein abundances of AMP-activated protein kinase α (AMPK), mammalian target of rapamycin (mTOR) and microtubule-associated protein 1 light chain 3β (LC3-I and II isoforms) in *longissimus* and *semispinalis* muscles of low (LRFI) and high (HRFI) residual feed intake (RFI) pigs raised in good or poor hygiene conditions.

Item	LRFI		HRFI		SEM	*P* values		
	Good	Poor	Good	Poor		Hyg	Line	Line x Hyg
N	9	10	7	6				
*Longissimus*								
Total AMPK, AU	1.47	1.63	1.93	1.97	0.42	0.47	0.01	0.69
pAMPK, AU	0.40	0.38	0.43	0.49	0.10	0.56	0.07	0.34
pAMPK/AMPK	0.27	0.24	0.23	0.25	0.05	0.83	0.44	0.24
Total mTOR, AU	1.59	1.40	1.50	1.72	0.31	0.89	0.36	0.09
pmTOR, AU	1.61	1.47	1.43	1.54	0.31	0.89	0.63	0.26
pmTOR/mTOR	1.01	1.05	0.95	0.94	0.14	0.68	0.10	0.58
LC3-I, AU	4.61	4.81	4.49	5.03	1.75	0.57	0.94	0.79
LC3-II, AU	2.89	3.64	2.13	4.19	1.84	0.04	0.89	0.34
LC3-II/LC3-I	0.66	0.82	0.53	0.93	0.45	0.10	0.97	0.46
*Semispinalis*								
Total AMPK, AU	1.60	1.66	1.35	1.53	0.16	0.04	0.003	0.32
pAMPK, AU	3.08	3.81	2.22	3.18	0.96	0.02	0.04	0.74
pAMPK/AMPK	1.93	2.30	1.66	2.09	0.59	0.07	0.25	0.87
Total mTOR, AU	2.73	3.01	2.43	2.91	0.40	0.01	0.19	0.49
pmTOR, AU	4.10	4.72	3.96	4.26	1.06	0.20	0.44	0.68
pmTOR/mTOR	1.54	1.59	1.63	1.44	0.39	0.59	0.84	0.40
LC3-I, AU	8.34	9.77	10.45	9.33	2.35	0.86	0.34	0.15
LC3-II, AU	2.71	2.93	2.27	3.02	1.52	0.38	0.75	0.64
LC3-II/LC3-I	0.33	0.31	0.25	0.36	0.17	0.44	0.84	0.29

Proteins were quantified by western blot and expressed as arbitrary units (AU). pAMPK refered to Thr172 phosphorylated AMPKα and pmTOR to Ser2448 phosphorylated mTOR. Values are LS means.

## Discussion

The current study revealed that the decrease in growth performance (higher ADFI and lower ADG and G:F) observed after a 6-week exposure of pigs to poor hygiene of housing conditions was associated with muscle-dependent changes in energy metabolism in both RFI lines. Based on previous observations, it has been suggested that there may be some differences in the use of energy between the two lines^27^. Nevertheless, most of the investigated parameters related to metabolic pathways did not differ between the two lines in the current study. Besides, with the exception of two parameters (glycolytic potential in SM and lactate dehydrogenase activity in LM), there was no significant interaction between hygiene of housing conditions and RFI lines.

The increase in ADFI and the decrease in ADG induced by the exposure of pigs to poor hygiene conditions are consistent with the changes reported from a larger set of animals^7^. The concentrations of several hormones were assessed to clarify the mechanisms underlying the impact of hygiene of housing conditions on growth and metabolic parameters in both lines. Only plasma IGF-I concentration were affected in our experimental design, with lower IGF-I concentration in pigs exposed to poor hygiene conditions. This is consistent with previous studies showing a reduced plasma IGF-I concentration in pigs submitted to a LPS challenge^28,29^ or in pigs with lower growth performances in others studies^30,31^. Irrespective of housing conditions, lower plasma IGF-I concentrations were also measured in HRFI pigs compared with LRFI pigs. Previous studies have observed higher IGF-I circulating concentrations in HRFI pigs compared with LRFI pigs considered around post-weaning^32,33^, whereas no differences were reported between the two RFI lines in older pigs including pigs at market weight^34–36^. Previously, plasma leptin concentrations have been shown to be slightly higher in HRFI than in LRFI pigs^34^, but others did not report any significant differences between the two lines^35^. The discrepancies between studies may be due to differences in pig nutritional status^35,37^.

Skeletal muscles account for approximately 50% of the body mass, so that any changes in their metabolic features may have major consequences at the whole animal level. The two investigated muscles are of different contractile and metabolic types^38^, with LM being a fast-twitch glycolytic muscle involved in movement, and SM being a predominantly slow-twitch oxidative muscle involved in posture with chronic contractions to support the head. Importantly, we showed that a shift towards a more oxidative and less glycolytic metabolism occurred in the SM when pigs of both RFI lines faced poor hygiene of their housing conditions. This was highlighted by increased activities of mitochondrial enzymes (CS, an enzyme of the tricarboxylic acid cycle and HAD, a key enzyme in fatty acid oxidation) and level of COX IV. Oxidative changes in LM were moderate and differed only between hygiene conditions in the HRFI line. These results are consistent with other studies showing that changes in feed consumption and growth rate of pigs modify muscle metabolism according to muscle type^31^. For instance, reduced growth rate induced by dietary deficiency in sulphur aminoacids decreased glycolytic metabolism in LM and increased oxidative metabolism and capacity to oxidize fatty acids in *rhomboideus* muscle, a mixed slow and fast-twitch oxidative muscle^20,39^. The current study also indicates an activation of the AMPK pathway in SM in response to hygiene degradation of pig housing conditions. This change is consistent with the fact that AMPK plays an important role in mitochondrial biogenesis and in the regulation of fatty acid oxidation and transport^40,41^. The greater use of fatty acids for oxidation pathway in the SM of pigs housed in poor hygiene conditions could explain the lower circulating concentrations of free fatty acids reported in poor versus good hygiene conditions for pigs at the end of the sanitary challenge^7^, considering that a significant part of the fatty acids used by myofibers originate from the blood stream. Those plasma free fatty acids may be provided by lipolysis of fat depots, in particular at the perirenal location, as suggested by Sierzant et al.^8^ who reported a reduction of the proportion of perirenal adipose tissue in pigs raised in poor vs good hygiene conditions from the same experiment. A shift towards increased fatty acid oxidation might be a strategy to spare glucose and suggests the reallocation of energy-yielding nutrients to support the immune response.

Muscle protein synthesis has been described as muscle-specific in pigs dealing with a septic challenge^42^. In muscle, the mTOR pathway plays a crucial role in the regulation of the major cellular processes such as protein synthesis, and involves the phosphorylation of mTOR at Ser2448^43^. In SM, total mTOR was greater in pigs of the two RFI lines when housed in poor hygiene conditions, but there were no changes in pmTOR levels or pmTOR/mTOR ratio, a marker of the mTOR pathway activation. A recent study based on severe viral challenge inducing BW loss in post-weaned growing pigs reported that the mTOR signaling cascade was inhibited^16^. The discrepancy between our study and the aforementioned one could be related to the severity of the immune challenge. Indeed, animals lose BW in Helm et al.’s study, whereas animals housed in poor hygiene conditions still gained BW but less than in good hygiene conditions in our study.

Because the autophagic-lysosomal pathway is a major proteolytic system, we investigated the conversion of LC3-I into LC3-II (a lipidated form of LC3-I incorporated into the autophagosomes) used as a marker of autophagic pathway activation^44^. As observed for energy metabolic pathways, hygiene of housing conditions induced muscle-specific changes in this marker of autophagy. Noteworthy, we found an activation of the autophagic-lysosomal system (increased abundance of LC3-II) in LM of pigs raised in poor hygiene conditions compared with pigs raised in good conditions, whereas no changes were identified in the SM. These data confirm that the autophagy pathway is differently activated according to muscle metabolic type and physiological function. Indeed, studies performed in mice in response to pulmonary infection or starvation, consistently reported greater LC3-I lipidation in muscle having a low oxidative capacity than in muscle with a high oxidative capacity^45,46^. Irrespective of hygiene conditions, LC3-I level (autophagic pathway) as well as mTOR, pmTOR and the pmTOR/mTOR ratio (protein synthesis pathway) were greater in SM than in LM (statistical analyses not shown), suggesting greater protein turnover in SM in accordance with data comparing slow- and fast-twitch skeletal muscles in different species^47–49^. One of the strongest autophagy stimuli is food deprivation. In our study, autophagy seemed to be activated in pig muscle in response to the hygiene stress but this was associated to an increased rather than a decreased food intake of pigs. Therefore, further work is needed to elucidate the specific roles of autophagy on skeletal muscle growth in pigs.

In conclusion, the current study revealed that degradation of hygiene conditions during pig rearing affected skeletal muscle metabolism in a muscle-type dependent manner. The shift toward a more oxidative energy metabolism in SM and autophagy stimulation in LM underlined different metabolic strategies at the muscle level to cope with poor hygiene conditions of pigs and supply the immune system.
